# Enhancing health and therapeutic potential: innovations in the medicinal and pharmaceutical properties of soy bioactive compounds

**DOI:** 10.3389/fphar.2024.1397872

**Published:** 2024-10-03

**Authors:** Ubaidur Rahman, Zohaib Younas, Ilyas Ahmad, Tayyaba Yousaf, Rafia Latif, Ume Rubab, Hira Hassan, Unsa Shafi, Zia-ur-Rehman Mashwani

**Affiliations:** Department of Botany, PMAS Arid Agriculture University, Rawalpindi, Punjab, Pakistan

**Keywords:** soybean, pinitol, isoflavones, therapeutic, cancer, diabetes, peptides

## Abstract

An extensive examination of the medical uses of soybean bioactive components is provided by this thorough review. It explores the possible health advantages of isoflavones with phytoestrogenic qualities, like genistein, which may lower the risk of cancer. The review highlights the different roles and possible anticancer activities of phenolic compounds, phytic acid, protease inhibitors, lignans, and saponins, among other bioactive components. It also addresses the benefits of dietary fiber and oligosaccharides derived from soybeans for intestinal health, as well as the impact of soy protein on diabetes, obesity, cancer, and cardiovascular health. Conjugated linoleic acid (CLA) has anticancer and cholesterol-lowering properties; its involvement in promoting metabolic processes is also examined. Pinitol is highlighted in the study as a blood sugar regulator with promise for controlling insulin signaling. In this review, we aim to affirm soybeans’ potential as a high-functional, well-being food by examining their recently discovered therapeutic and pharmacological capabilities, rather than to improve upon the previous studies on the reported nutritional advantages of soybeans.

## 1 Introduction

*Glycine max* or soybean, is a noteworthy subtropical food crop that is prized for its high-quality oil and protein-rich composition, which contribute to its economic significance. Soybeans are widely available and used over the world as a grain and as the main source of raw materials for the production of soymilk and tofu (soybean curds). Soybeans are a Chinese crop that have been cultivated for millennia. They are ranked among the top five main plant foods in ancient Chinese history, along with barley, millet, wheat, and rice (Lee et al., 2020; Swallah et al., 2023). Soybean processing produces a number of useful products, including as oil, bran, flour, soluble extract, and textured protein. In spite of these potential benefits, soybeans have not received enough attention in industrial products and human diets (Swallah et al., 2023). Nonetheless, as more people become aware of soy and its benefits for their health, soy products are becoming more widely available in the functional food market (Feng et al., 2021). This increase in demand is in line with the growing understanding that plants are abundant in phytochemicals, bioactive, non-nutritive, and perhaps beneficial compounds (Swallah et al., 2021). This paradigm shift has led to the emergence of the concept of “functional foods.”

In these goods, substances originating from plants are added to various forms in an effort to provide advantageous qualities and functions to the body. Soybean represents the confluence of nutritional value and health-promoting characteristics, having been one of the first foods generally recognized for such advantages (Swallah et al., 2023).

Recent research has generally ignored other features of soybeans, such as their nutritional value, in favour of examining the potential medicinal and therapeutic uses of soy bioactive compounds. From a nutritional perspective, foods made from soy have been demonstrated to be beneficial in treating a variety of lifestyle disorders (Wu et al., 2017). In addition to improving bone health, these nutrients can reduce the risk of prostate, colorectal, and breast cancers, among other cancers. Additionally, eating soy has been associated with a lower risk of cardiovascular diseases, type II diabetes, obesity, cognitive decline, renal dysfunction, menopausal symptoms, atherosclerosis, and coronary heart disease, partly because it lowers low-density lipoprotein levels (Xiao, 2011). Recent research suggests that soy consumption may inhibit the activity of delta-6 desaturase (D6D), an essential enzyme involved in the endogenous synthesis of long-chain polyunsaturated fatty acids (LC-PUFA), despite the well-documented effects of increased soy consumption on triglycerides and cholesterol (Gonzalez-Soto et al., 2021). This nuanced perspective highlights the diverse nutritional possibilities of soy-based foods and how they can contribute to overall health promotion in addition to their well-known medicinal and therapeutic advantages.

The primary objective of this review is to systematically compile evidence-based data regarding the significance of bioactive compounds present in soybeans for human health. This compilation aims to explore the pharmacological and therapeutic potential of these materials, perhaps leading to future applications in both industrial and clinical settings.

## 2 Bioactive profiles of soybean

Foods typically have trace levels of bioactive compounds, which are dietary components that may be used to treat oxidative stress, metabolic disorders, and reduced pro-inflammatory states (Swallah et al., 2020). Researchers are constantly monitoring these compounds to assess potential health impacts as shown in Table 1 (Ng et al., 2013). Soybeans have a wide spectrum of bioactive macromolecules, which have been connected to several health advantages. Frequent consumption has been linked to a decreased risk of several illnesses, such as osteoporosis, cardiovascular disease, cancer, and cognitive impairment. It has also been linked to menopausal symptoms.

**TABLE 1:** Literature review on therapeutic potential of some major isoflavone and other phytochemicals extracted from soybean.

Bioactive	Dose	Model	Major effects	Results	References
Gaidzein	Genisten at 100 µm	*In vivo* female ICR model	Improved DSS, Expression of IL-1β, IL-6, IL-7, and TNF-α	Isoflavone mediated DSS induced inflammation, linked to improved antioxidant functioning along with TLR4/MyD88 inhibition	Noda et al. (2012)
Genistein	50–100 µm	*In vitro* model	Elviation of SOD, CAT and GSH lvels along with reduced LPS induced NO production	Modulation of inflammatory response via inhibition of PGE2 and NO along with modulation of vital genes	Blay et al. (2010)
Genistein	100 mg/kg of body weight	*In vivo* wistar rat model	Reduction in colonic COX-1 expression and MPO activity	Oral administration of genistein showed anti-inflammatory response	Seibel et al. (2009)
Genistein	200 µm	*In vitro*	TLR mediated IL-6 prudction DNA binding and upregulation of p53 protein	Reduced IL-6 mRNA production along with inhibtry effects for p53	Dijsselbloem et al. (2007)
Genistein mixed with diet	100 mg/kg	*In vivo*	Lowered colonic weight, rectal bleeding and diarrhoea ratio	Alleviation of DSS- caused injury, inflammation and gut dysfunction	Zhang et al. (2017)
Morphine-5	10 mg/kg/day	*In vivo*	Activation of adiponectin and stimulation of peroxisomes proliferator	Reduction of blood glucose levels	Yamada et al. (2012)
SFPs	—	*In vivo*	Inhibition of dipeptidyl peptidase, salivary alpha-amylase and intestinal alpha-glucosidase	Regulation of postprandial glucose response	González-Montoya et al. (2018)

**FIGURE 1:**
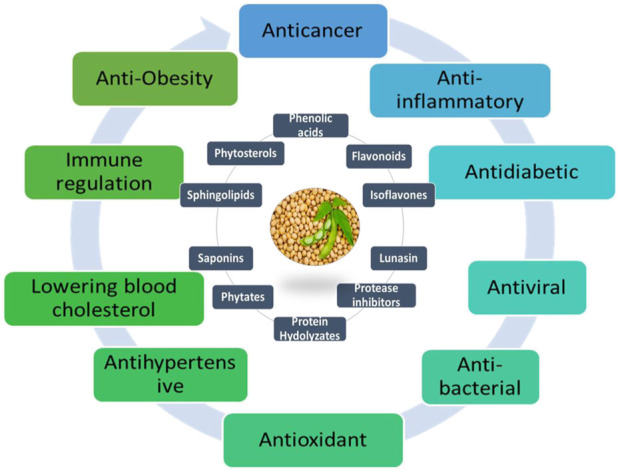
Major therapeutic bioactive compounds and disease targets of soybean.

The primary bioactive components of soybeans are phytosterols, isoflavones, proteins or peptides, saponins, carotenoids, tocopherols, and protease inhibitors, according to Chatterjee et al. (2018); Wu et al. (2017). Additionally, soybeans contain other constituents, including but not limited to glycinin, Kunitz trypsin inhibitor, Bowman-Birk inhibitor, peroxisomal proteins, hemagglutinin, SbPRP protein, neutral PR-5 protein, ferritin, isoflavone-deprived soy peptide, defense proteins (such as β-glucan-binding protein, calmodulin, glysojanin, lunasin, and disease resistance protein), enzymes (tyrosine ammonia-lyase and phenylalanine ammonia-lyase), defensive enzymes (cysteine proteinase, isocitrate lyase, isoflavone synthase, chalcone reductase, and vestitone reductase), UDP-glucose (including betaglucosidase, flavonoid 3-O-glucosyltransferase, 5′-adenylylsulfate reductase, isoflavone conjugate-hydrolyzing β-glucosidase, ATP sulfurylase, and 2-oxoglutarate-dependent dioxygenases genes), polysaccharides, glyceollins, anti-carcinogenic daidzein-rich fraction, and isoflavones (Ng et al., 2013). Soybeans’ wide variety of bioactive compounds emphasizes both its intricate nutritional composition and potential health advantages as shown in Figure 1.

## 3 Phenolic compounds

Tannic acid, flavonoids, phenolic acids, and hydroxycinnamic acid derivatives are examples of phenolic chemicals, often known as phenolics, which are antioxidants present in the majority of plants (Król-Grzymała and Amarowicz, 2020). Tannic acid, which is naturally occurring as gallic acid, is a polyhydroxyl phenol ester (Easwar Rao and Viswanatha Chaitanya, 2020). Moreover, soybeans contain isoflavone, a byproduct of flavonoids and phenolic acids (Li et al., 2011).

### 3.1 Isoflavones

In addition to lignans, isoflavones are phytochemicals generated from plants that are activated by the gut flora and have physiological activity comparable to that of estrogen (Hu et al., 2020). Classify them as phytoestrogens (Hu et al., 2020). Classified the various isoflavones present in soybean hypocotyls into four classes according to their chemical structures: i) aglycons (daidzein, genistein, and glycitein); ii) glycosides (daidzin, genistin, and glycitin); iii) acetyl glycosides; and iv) manonyl glycosides. Isoflavones have been shown in numerous studies to activate estrogen receptors in the vagina, oocytes, and mammary glands; in addition, depending on their physiological environment or chemical makeup, they may have estrogenic or antiestrogenic actions (Nakai et al., 2020). As an antiestrogen, isoflavone, for example, has been associated with a lower incidence of prostate and breast cancers and, both *in vivo* and *in vitro*, has antioxidant properties similar to those of vitamin C and E (de Jesus et al., 2018; Sivoňová et al., 2019). Moreover, tyrosine protein kinase is inhibited by the isoflavone that an oncogene produces (Aichinger et al., 2016). According to research by Benkerroum (2020), genistein stands out among soybean isoflavones in that it has the ability to successfully inhibit the growth of cells associated with cancers of the breast, colon, lung, prostate, and skin *in vitro*. Furthermore, by blocking vasculogenesis, which halts the passage of oxygen or nutrients, genistein inhibits the formation of boils (Martínez-Poveda et al., 2019).

Ziaei and Halaby (2017) found that studies on the impact of isoflavone consumption on the menstrual cycle in Western women reduce the incidence of breast cancer. Furthermore, isoflavones have a slight estrogenic effect that reduces menopausal symptoms without having a negative impact on health (Ahsan and Mallick, 2017). Isoflavones show promise as cholesterol-lowering medicines because they have the ability to cut blood cholesterol by up to 35% as shown in Table 1 (Taku et al., 2007). On the other hand, animal protein called casein has been linked to elevated blood cholesterol levels (Koury et al., 2014). When people consume insufficient amounts of protein, their stored fat is converted to protein, which raises blood fat levels and blood cholesterol (Ramdath et al., 2017).

**FIGURE 2:**
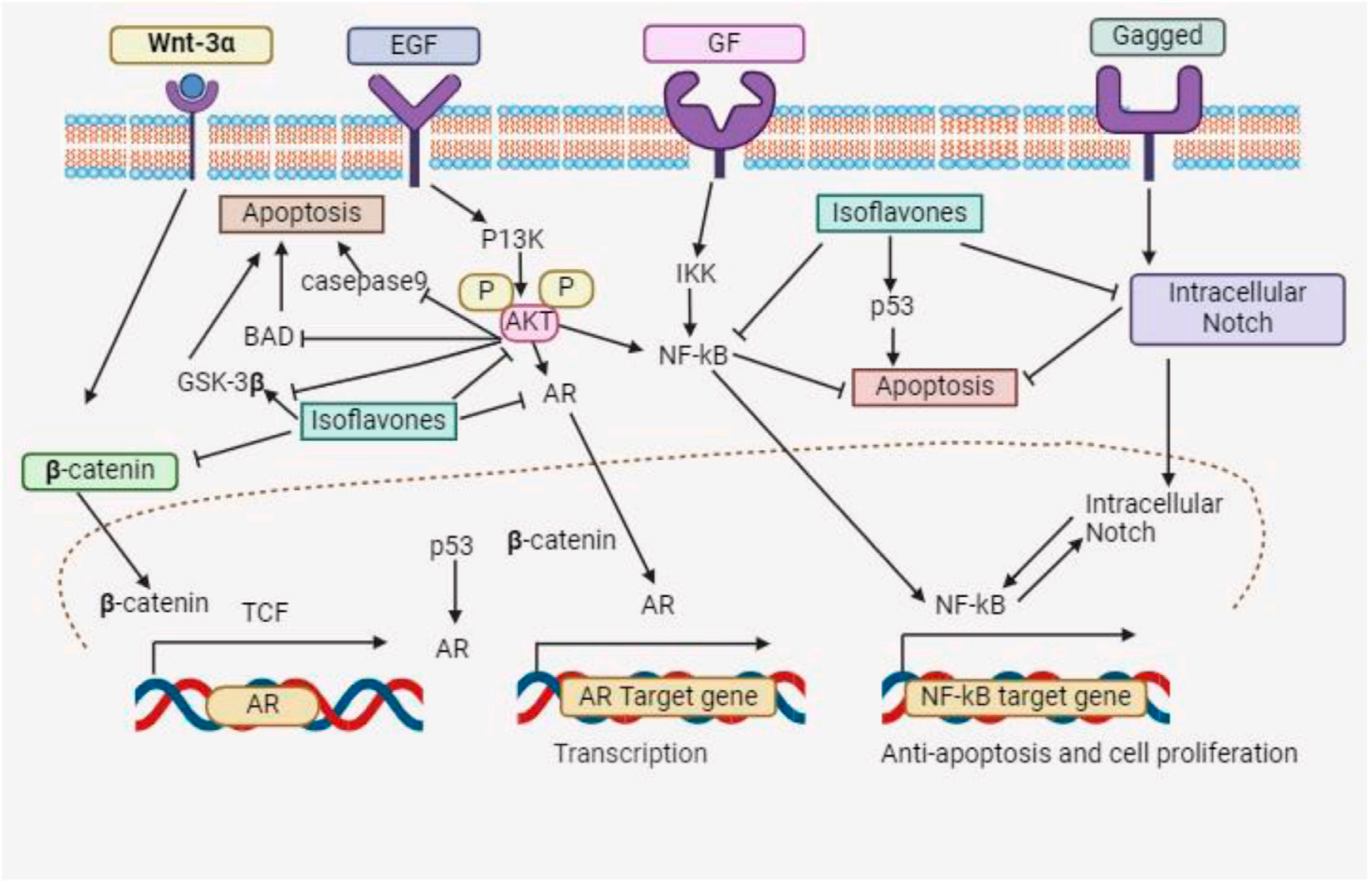
This schematic diagram shows different pathways which indicate that the isoflavones have the therapeutic potential to induced cancer cell death.

About 15% of menopausal women in the US undergo estrogen therapy, a practice linked to a higher risk of malignancies of the reproductive organs (Palacios et al., 2019). Due to its ability to replace estrogen in this population, soybeans a natural dietary source are becoming more and more popular as an alternative (Messina, 2016). By boosting vitamin D activity, decreasing calcium loss from bones, and improving calcium absorption, estrogen plays a critical role in lowering the incidence of osteoporosis (Dall and Britt, 2017). Phytoestrogens are the isoflavones found in soybeans that are structurally and functionally similar to estrogen (Li et al., 2011). According to Tuli et al. (2019), isoflavones, in particular genistein, have significant anticancer properties. They promote normal cell division while suppressing the division of cancer cells by weakly attaching to estrogen receptors. In contrast to the frequent adverse effects of estrogen therapy, isoflavones reduce menopausal hot flashes without causing hyperlipidemia or altering the muscle layers in the breast and uterus (Ahsan and Mallick, 2017). In addition, isoflavones counteract osteoporosis, a common issue among elderly women, by increasing bone density and preventing bone reabsorption (Akhlaghi et al., 2020). Figure 2 summarizes the several physiological roles that isoflavones play.

### 3.2 Phenolic acids

Eight phenolic acids, including p-hydroxy benzoic acid, vanillic acid, gentisic acid, salicylic acid, chlorogenic acid, and cinnamic acid (Ferreira et al., 2019), are abundant in soybeans. Notably, browning—an unwanted dietary impact that depletes nutrients and modifies color and flavor—can arise from the hydrolysis of chlorogenic acid to caffeic acid (Liu et al., 2019). However, according to Gao et al. (2017), both caffeic acid and chlorogenic acid have the ability to prevent the development of nitrosamines both *in vitro* and *in vivo*. Moreover, these phenolic acids show that they can stop the rat liver’s production of aflatoxin B1 (Benkerroum, 2020). Phenolic acids, which function as antioxidants, can also prevent reactive oxygen species from damaging DNA (Kiokias et al., 2020). These phenolic acids have a dual character that draws attention to both their possible detrimental effects on food quality and their beneficial effects on increasing antioxidant activity and thwarting damaging processes.

## 4 Phytic acid

Phytic acid is composed of six phosphate groups that are symmetrically connected to a myo-inositol ring. It is sometimes referred to as myo-inositol hexaphosphate (IP6) (Silva and Bracarense, 2016). Although found (Gao et al., 2017) in many plants, phytic acid is particularly prevalent in grains and legumes; 2.58% of soybean seeds have phytic acid in them (Hummel et al., 2020). Phytic acid hydrolyzes during food processing to produce myo-inositols with fewer phosphate groups, IP1, IP2, and IP3 (which contain one, two, and three phosphate groups, respectively) (Gupta et al., 2015).

Humans excrete 0.5–0.6 mg/L of phytic acid in their urine, which represents 1–3 percent of the total amount consumed (Marolt and Kolar, 2020). Phytic acid, which is widely distributed in the outer shell of grains and beans, chelates with divalent ions such as Fe2+, Zn2+, Mg2+, and Ca2+ to prevent absorption in the small intestine (Gupta et al., 2015). Phytic acid also functions as a non-nutritional ingredient, preventing the body from using minerals and blocking vital digestion enzymes like α-amylase, trypsin, and pepsin by adhering firmly to the protein base (Popova and Mihaylova, 2019). Phytic acid was once thought to be non-nutritional because of its influence on mineral absorption, but more recently, its antioxidant, anticancer, and lipid-lowering properties have made it popular (Abdulwaliyu et al., 2019) Figure 3.

**FIGURE 3:**
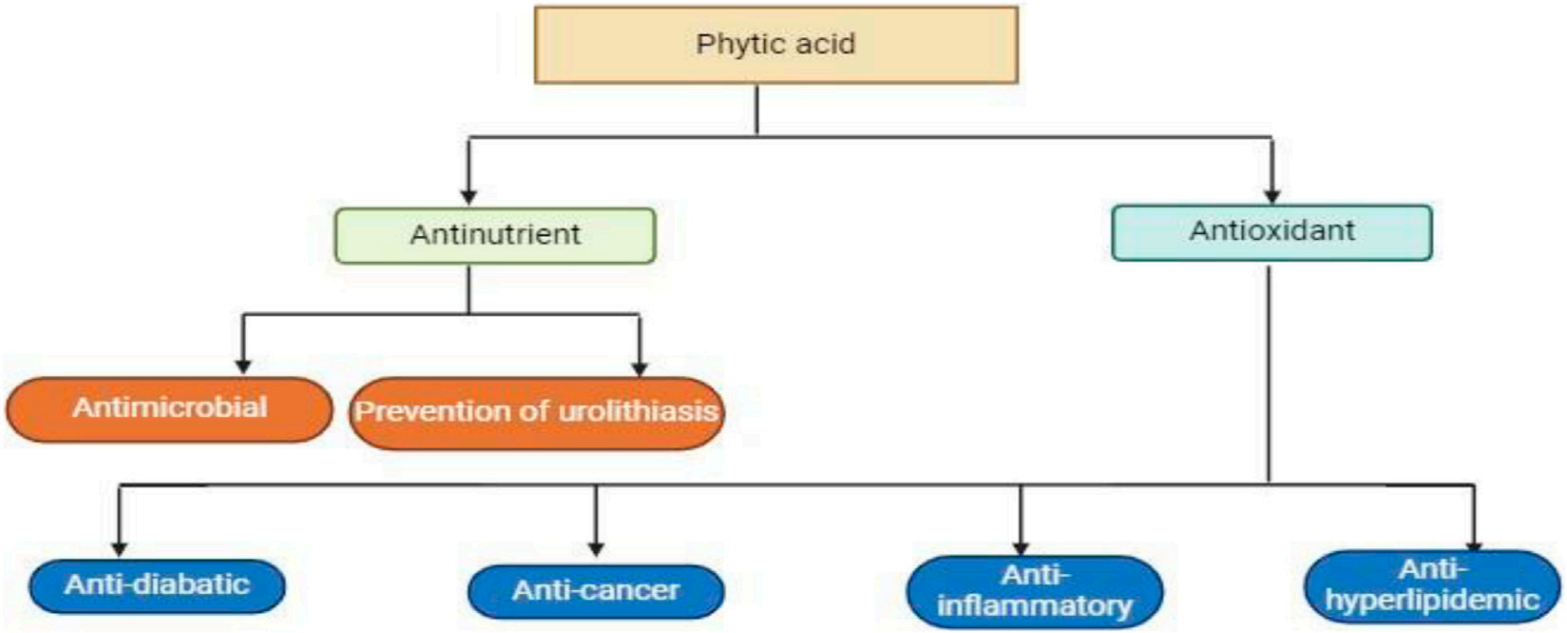
This diagram show that Phytic acid have the therapeutic potential to decreased and control the following mentioned diseases in the figure.

The storage of cations and phosphorus is one of the biologically active functions of phytotic acid (Kumar et al., 2021; Zhao, 2019) report evidence of iron-induced oxidative damage occurring *in vivo*, wherein iron can cause lipid or cell oxidation through the formation of hydroxyl radicals. Conversely, phytic acid can bind to iron, which prevents hydroxyl radical production and halts cellular oxidation (Abdulwaliyu et al., 2019). One way that this antioxidant effect is being studied in relation to food processing is through studies on the delivery of phytic acid to minimize oxidation during food processing.

Furthermore, because phytic acid activates the expression of tumor-suppressor genes including p53 and WAF-1/p21, those who consume more grains and vegetables high in phytic acid had a decreased risk of colorectal cancer. By inhibiting the growth of cancer cells and encouraging cell differentiation, phytonic acid also has anticancer action (Vucenik et al., 2020). Furthermore, lower inositol phosphates, such as IP3 and IP4 (which have three and four phosphorus groups, respectively), influence the body’s communication systems and have a major biological role in controlling cell-to-cell responses (Mukherjee et al., 2020).

## 5 Protease inhibitors

Soybeans and other plant systems such as grains, grass, potatoes, fruits, vegetables, peanuts, and corn contain protease inhibitors (PIs) (Srikanth and Chen, 2016). According to Hellinger and Gruber (2019), soybeans include Kunitz and Bowman-Birk types of PIs that block the actions of chymotrypsin, elastase, and serine proteases. Soybean PIs, which were once thought to be antinutritional inhibitory factors, have drawn interest lately due to their possible anticancer effects (Gitlin-Domagalska et al., 2020).

Trypsin inhibitors have been demonstrated to suppress the production of free radicals, preventing cells from suffering oxidative damage, which is the primary mechanism by which PIs contribute to health benefits (Srikanth and Chen, 2016). In particular, the Bowman-Birk type PI, which is well-known for its chymotrypsin inhibitory effect, inhibits the function of the tumor promoter 12-o-tetradecanoylphorbol-13-acetate, which in turn inhibits the expression of the oncogene MYC, decreases hydrogen peroxide production, and protects DNA’s helical structure while preventing DNA oxidation (Gitlin-Domagalska et al., 2020).

**FIGURE 4:**
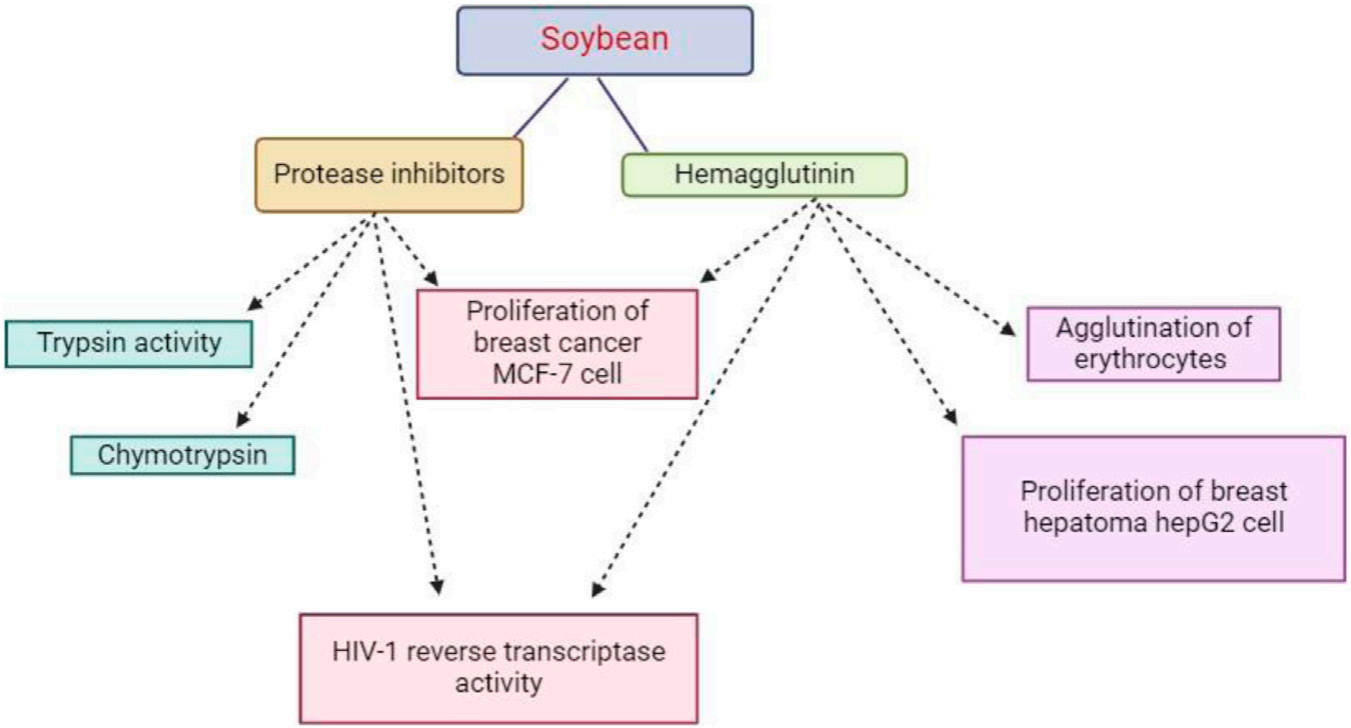
This diagram indicate that protease inhibitors have some pharmacological and therapeutic activities against some diseases.

Notwithstanding structural variations, recent studies have demonstrated the anti-carcinogenic properties of compounds present in other plants, including soybean PIs, retinoids, garlic acid, nicotinic acid, tamoxifen, and epigallocatechin gallate. These compounds work by inhibiting the generation of superoxide radicals or H2O2 by tumor promoter factors, as shown in Figure 4. Moreover, trypsin inhibitors found in soybeans facilitate the release of insulin, which controls blood sugar levels (Cristina Oliveira de Lima et al., 2019).

## 6 Lignans

Lignans, which are found in plants in small amounts, help to build the framework of the cell wall when they are bound. After consumption, intestinal bacteria transform them into enterolactone or enterodiol, which are then eliminated in the urine as conjugates of glucuronides (Frezza et al., 2020). Flax seeds and soybeans are good sources of lignans or lignan precursors. According to Rodríguez-García et al. (2019), lignans are categorized as phytoestrogens because they have chemical structures similar to those of estrogen and can control estrogen levels. According to research, a significant lignan consumption may reduce the body’s free estrogen content, which may reduce the risk of breast cancer caused by estrogen (Peterson et al., 2010).

**FIGURE 5:**
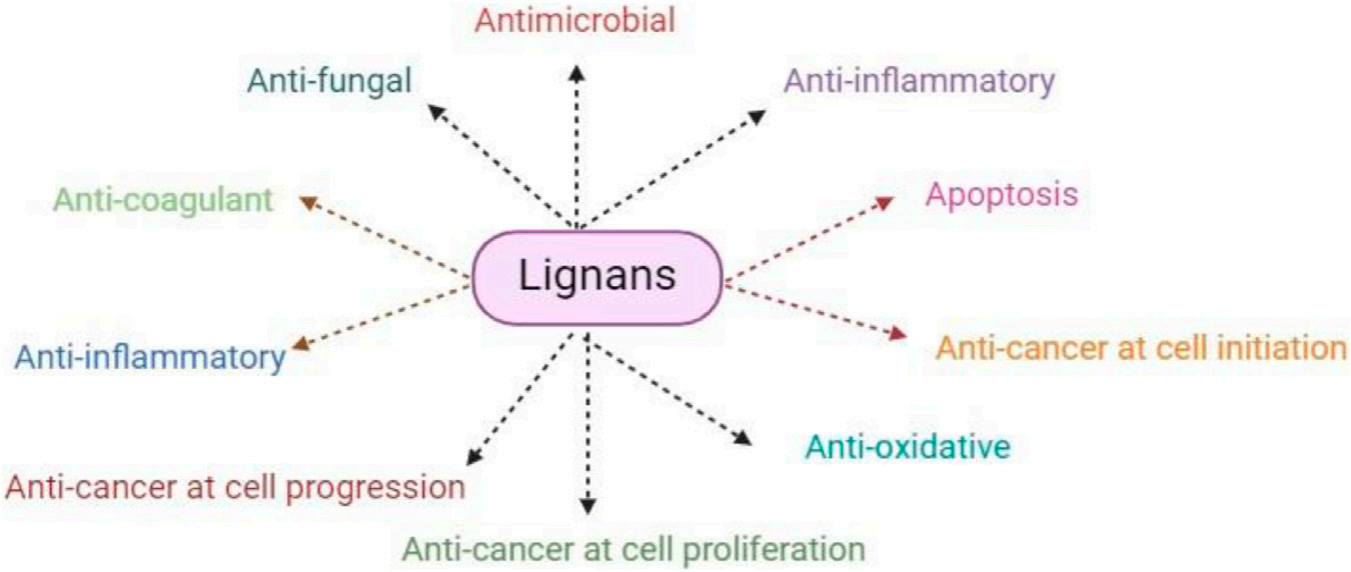
This figure explained the overall pharmaceutical and therapeutic potential of lignans against different diseases and pathogens.

Regarding (Vinardell and Mitjans, 2017), there is evidence that suggests lignins can stop breast cancer cells from proliferating in tissue culture systems. To be more precise, lignans block the action of 7-α-hydroxylase, which helps to produce bile acid from cholesterol, or they block the activity of 5α-reductase and 17β-hydroxysteroid dehydrogenase, which are involved in estrogen biosynthesis and metabolism. This combined effect may reduce the incidence of colon cancer and cancer linked to sex hormones, respectively (Chang et al., 2019). Furthermore, by working in concert with flavonoids and other phytochemicals, the ingestion of foods high in lignan can strengthen their anticancer qualities which are given in Figure 5 (Rodríguez-García et al., 2019).

## 7 Saponin

According to Shi et al. (2004), out of all the edible legumes, soybeans have the greatest saponin content. According to Moses et al. (2014), saponins that include a covalently linked non-saccharide can be classified as either triterpene or steroid saponins. Based on the skeleton of the non-saccharide section, triterpene saponins are further classified as oleanane, ursane, dammarane, and cycloartane (Vincken et al., 2007; Kamo et al., 2014) Eleven distinct saponins, six from group A and five from group B, are extracted from the soybean hypocotyl (Neacsu et al., 2020).

L-arabinose, L-rhamnose, D-xylose, D-glucuronic acid, D-galactose, and D-glucose are among the monosaccharides of soyasaponins. The hypocotyl and germ layer contain high concentrations of saponins, whereas the outer skin contains none at all (Lim, 2012; Kamo et al., 2014) states that the type and amount of soyaponins vary among species, with group B saponins ranging from 0.26% to 2.75% and group A saponins between 0.36% and 0.41%. Furthermore, during germination, the concentration of group B saponin increases (Guajardo-Flores et al., 2012). The amount of saponin is decreased when microorganism enzymes ferment, yet there is little data on how heating or processing affects saponin content (Tangyu et al., 2019).

Recent studies have reevaluated saponin, which was previously thought to be a bitter, non-nutritional chemical. These studies have shown saponin’s physiologically active roles. This puts it in the forefront as a functional nutrient and includes immune system stimulation, cholesterol lowering, and anticancer benefits (Zaynab et al., 2021). Particularly, soybean saponin shortens the duration of exposure to the mesentery, which speeds up the absorption of hazardous chemicals and lessens their toxicity (Tian et al., 2018). Because soybean saponin and cholesterol have similar molecular structures, they prevent cholesterol from being absorbed and promote its release (Ramdath et al., 2017).

Furthermore, saponin and vitamin E (tocopherol) work together to improve blood circulation and prevent skin imperfections (Choudhry et al., 2016). In addition to lowering blood levels of low-density lipoprotein (LDL, or “bad cholesterol”) and facilitating smoother blood flow, vitamin E also keeps brown spots, usually referred to as age spots, from developing on the faces of middle-aged and older people (Galmés et al., 2018).

**FIGURE 6:**
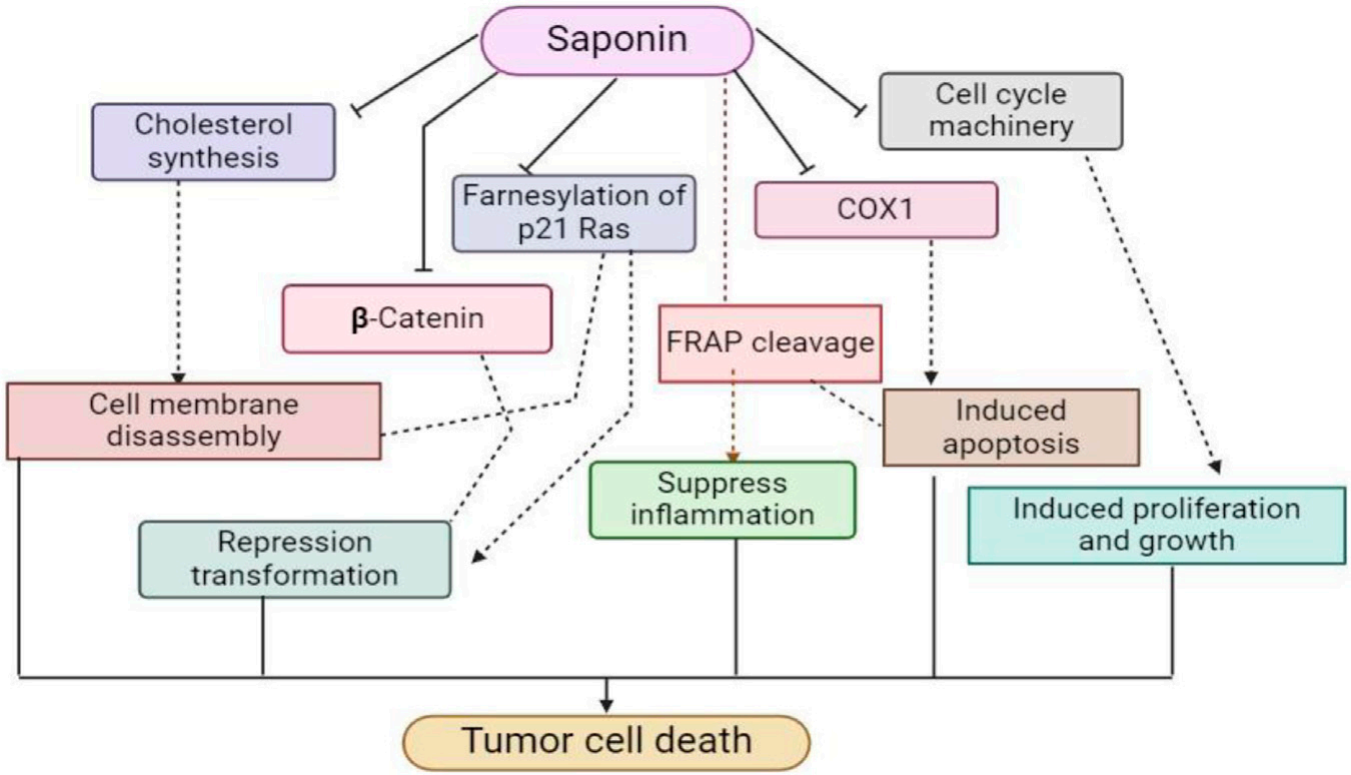
This schematic diagram shows the anti-cholesterol lowering mechanism and anti-cancer mechanism of saponin derivatives at cellular levels.

Moreover, saponins function as antioxidants similarly to phytic acids, preventing cell damage caused by free radicals (Ganesan and Xu, 2017). Notable is their ability to lower the rates of DNA mutations, especially in avoiding colon cancer (Tin et al., 2007). With a molecular composition similar to licorice saponins, soybean saponins may have anticancer properties that are being studied (Wang et al., 2019). Interestingly, saponins decrease DNA synthesis in tumor cells, increase killer cell activity, (Figure 6) function as a cell poison specific to sarcoma, and slow the growth of cervical and epidermal cancer cells (Fuchs et al., 2017). According to recent research, group B saponins from soybeans have an inhibitory effect on HIV infection (Li et al., 2018).

## 8 Dietary fiber and soy oligosaccharides

Dietary fiber, which is difficult for the body’s enzymes to digest, is found in large amounts in soybeans. Dietary fibers are classified as either water-insoluble (cellulose and lignin) or water-soluble (pectin and gum) (Dhingra et al., 2012). Colonic microbes degrade water-soluble dietary fiber to produce vital short-chain fatty acids such as propionic, butyric, and acetic acids. These fats help colonic cells absorb cholesterol and are essential nutrients for them (Markowiak-Kopeć and Śliżewska, 2020). However, by encouraging bowel movements and improving intestinal function, water-insoluble dietary fiber can avoid constipation (Bae, 2014).

According to Hussain et al. (2020), the cell wall of soybean shells is made up of pectin and insoluble fibers, with a high concentration of water-soluble fibers. Many physiological processes are made possible by the properties of dietary fiber, including as swelling, water retention, absorption of organic molecules, ion absorption and exchange, and breakdown by intestinal microbes (Rowland et al., 2018). One of the most significant benefits of soybean dietary fiber is its capacity to lower cholesterol (Ramdath et al., 2017). Additionally, as per (Axelrod and Saps, 2018), soybean fiber plays a crucial role in controlling constipation, promoting consistent bowel movements, and reducing the transit time of food through the intestines.

Approximately 4% stachyose and 1% raffinose make up the soluble oligosaccharides known as soybean oligosaccharides, which are found in soybeans. Although they are few in young plants, their concentrations greatly increase as the plants age. Humans cannot digest these oligosaccharides, which cause flatulence and encourage the large intestine to produce gas (Salarbashi et al., 2019). In spite of this, they have drawn notice for encouraging the development of advantageous bacteria in the intestines (Pan et al., 2018).

**FIGURE 7:**
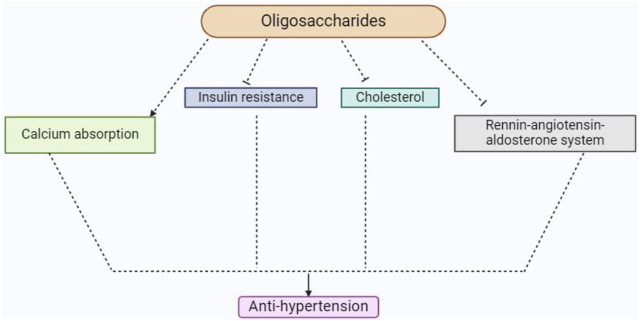
This diagram indicates the anti-hypertension activity of soybean oligosaccharides and their derivatives.

In addition to supporting vitamin synthesis in the gut, soybean oligosaccharides and dietary fiber also prevent the growth of pathogenic bacteria and the creation of amines and ammonia (Rowland et al., 2018). They function as growth promoters for Bifidobacterium, a beneficial bacterium that improves immunity, stimulates peristaltic movement in the gut, facilitates absorption and digestion, and reduces inflammation. In order to maintain intestinal pH, inhibit the growth of dangerous bacteria, enhance bowel movements, avoid constipation, and preserve intestinal function, Bifidobacterium creates lactic acid (Piqué et al., 2019). Furthermore, according to Rowland et al. (2018), Bifidobacterium lessens the generation of carcinogens like phenol, skatole, and indole, inhibits the absorption of hazardous substances like ammonia and H2S, and lessens the effects of insulin resistance and high cholesterol on hypertension (Figure 7).

## 9 Soy protein and peptides

As the main source of the protein that makes up a large amount of human diet, soybeans are a rich supply of vital and high-quality amino acids that come from plants (Singer et al., 2019). Consumption of soy protein has not kept pace with production, even after decades of research on its technological uses dating back to the 1950s (Kearney, 2010). According to Rizzo and Baroni (2018), soy protein is used in a variety of physiologically functional foods, including dairy products, meat alternatives, infant formulas, sports drinks, and fortified grain products. Soy protein has gained interest recently as a phytochemical because of its potential to cure diabetes, cancer, obesity, and cardiovascular disease (Forni et al., 2019).

Serum cholesterol levels, which are impacted by dietary lipids and protein, have a significant correlation with the development of arteriosclerosis (Liu et al., 2019). When it comes to lowering serum cholesterol levels, plant-derived protein works far better than its counterpart generated from animals. Particularly soy protein has been shown to have a cholesterol-lowering effect, thus it’s best to consume both forms of protein in moderation (George et al., 2020).

**FIGURE 8:**
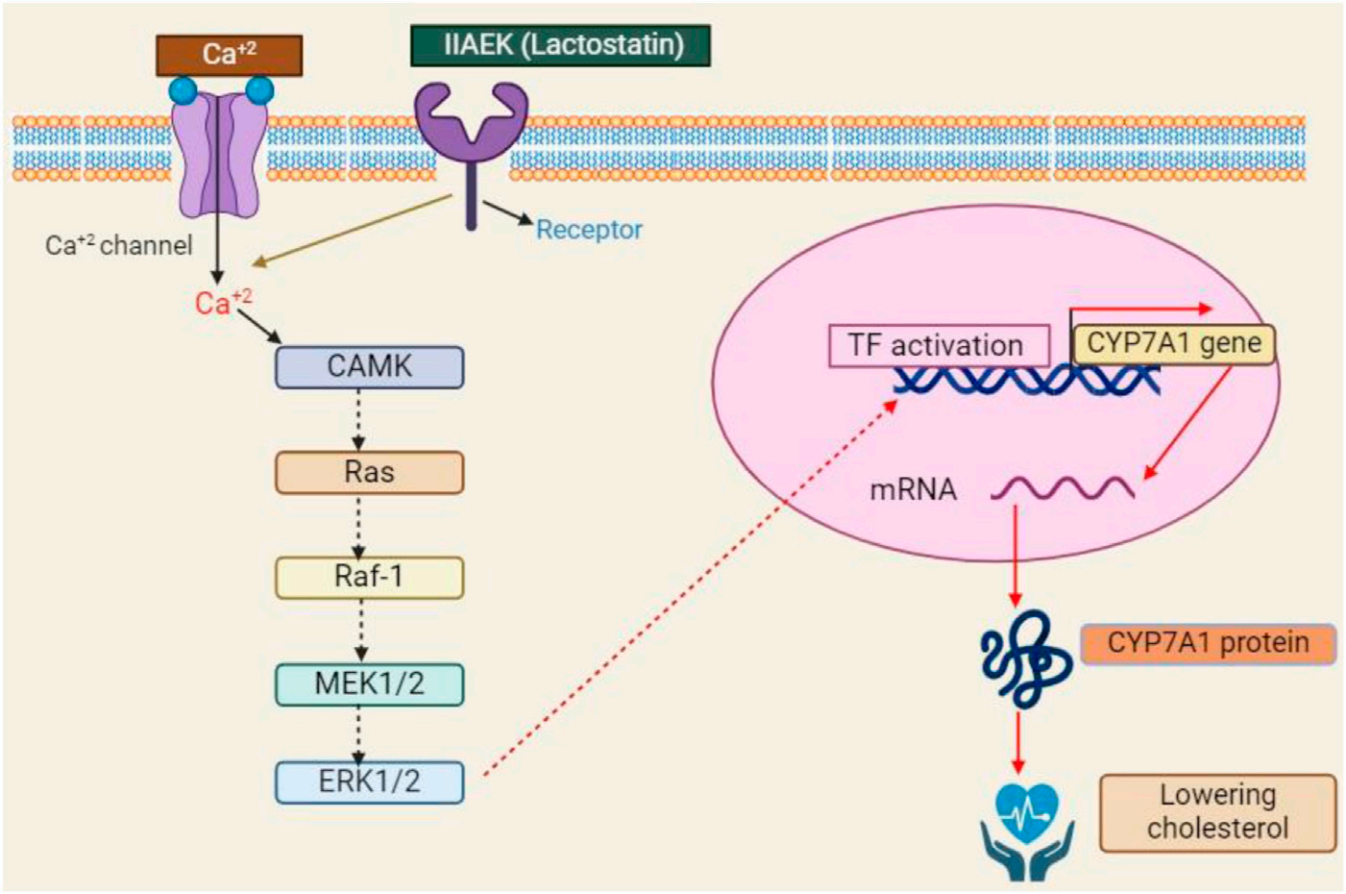
Cholesterol is the main cause of many diseases such as blood pressure and cardiovascular diseases, so this schematic diagram show that soybean proteins and peptides have the potential to lower the cholesterol level in the body.

Proteins are the source of bioactive peptides, which are essential for maintaining health because they are physiologically active and serve as a source of nutrition. Many soybean peptides have been discovered in the past 10 years, as Table 2 illustrates. According to Chatterjee et al. (2018), these peptides have a variety of characteristics, such as being hypocholesterolemic, anti-diabetic, hypotensive, capable of phagocytosing and boosting the immune system, anti-inflammatory, chemopreventive, and antioxidant. Consumer preference for items originating from natural sources is expanding, in part because bioactive peptides show fewer negative effects in humans when compared to medications and synthetic food additives (Sanjukta and Rai, 2016). Figure 8 show how soy protein reduced the cholesterol level.

**TABLE 2:** This table show different bioactive peptides of soy proteins and their therapeutical and pharmaceutical potential by using different types of models (Chatterjee et al., 2018).

Soy protein source	Bioactive peptides	Properties	Tested models
βCG	YVVNPDNDEN YVVNPDNNDEN LAIPVNKP LPHF	Hypocholesterolemic ACE inhibition	HepG2 human liver cell Assay for ACE inhibitory activity *in vitro*
βCG (α′-subunit)	Sulfur-13: MITLAIPVNKPGR Methylparaben: MITLAIPVN MITL is soymetide-4 KNPQLR; EITPEKNPQLR; RKQEEDEDEEQQRE	Immunostimulating FAS inhibitor	Phagocytosis assay FAS inhibition research; mouse adipocyte 3T3-L1
βCG (β-subunit)	YPFVV is soymorphin-5 YPFVVN is soymorphin-6	Anti-diabetic Immunostimulant	Diabetic KKAy mice High-level plus-maze examination in male ddY mice
Glycinin	IAVPGEVA IAVPTGVA LPYP SPYP HCQRPR	ACE inhibition Phagocytosis	ACE inhibitory assay Human polymorphonuclear leukocytes
Glycinin (A4 and A5)	LPYPR	Hypocholesterolemic	Mice given a 50 mg/kg dosage for 2 days
Lunasin	SKWQHQQDSCRKQKQ GVNLTPCEKHIMEKIQ GRGDDDDDDDDD	Anti-cancer Anti-oxidative Anti-inflammatory	MCF-7 breast cancer cells undergo TEN-mediated apoptosis; antioxidants in Caco-2 cells
Birk-bowman inhibitor		Chemoprevention by anti-cancer proteinase inhibition	Stop the production of ROS in PC-3, LNCaP, BRT-55T, and 267B1/Ki-ras prostate cancer cells
Defatted soy protein	X-MLPSYSPY	Anti-cancer	Stop P388D1 murine macrophages at the G2/M stage
Soy protein	YVVFK; IPPGVPYWT; PNNKPFQ; NWGPLV	Hypotensive	Spontaneously hypertensive rats
Genetically modified soybean protein	LLPHH; RPLKPW	Antioxidative; Antihypertensive	Hypertensive rats
Black soybean protein	IQN	Inhibition of adipogenesis	Mouse adipocytes 3T3-L1
Soymilk	RQRK; VIK	Anti-inflammatory	RAW 264.7 macrophages from mice
Fermented soybean	VAHINVGK	Inhibition of ACE	Assay for ACE inhibitory activity
Chymotrypsin Korean fermented soybean paste	HHL	Hypotensive	Spontaneously hypotensive rats
Soy sauce with fermentation	SY GY	Hypertensive	Rats with spontaneous hypertension

## 10 Lecithin

Complex lipids called lecithins are widely distributed in foods and tissues such as liver, brain, egg yolk, and soybean oil. They are essential to many metabolic functions. They help in the removal of waste products, aid in the absorption of fat-soluble vitamins and nutrients, and solubilize cholesterol to lower blood cholesterol levels (Li et al., 2017). Furthermore, studies on lecithin have demonstrated its efficacy in avoiding diabetes, preserving renal function, restoring liver function, and enhancing digestibility (Lecomte et al., 2017). Research has shown that adding lecithin to a low-fat, low-cholesterol diet will considerably raise HDL cholesterol levels while reducing LDL cholesterol by 15% when compared to a low-fat diet alone. Beyond its function in the body to dissolve, cleanse, and transport lipids, lecithin helps maintain skin health by clearing waste, triglycerides, and fatty materials from blood vessels (Van Hoogevest and Wendel, 2014). Moreover, lecithin functions as an antioxidant to reduce oxidative damage to vitamin A (Félix et al., 2020). According to Moré et al. (2014), it also has the beneficial impact of avoiding senile dementia and enhancing brain function.

Moreover, soybean lecithin is essential for stopping the brain’s acetylcholine from being reduced. Administration of lecithin has been shown to raise acetylcholine levels in several studies (including one with rats). Phosphatidylcholine has a beneficial effect on the cerebrum’s increased activity, which causes an increase in the consumption of acetylcholine. In addition to influencing lipid metabolism and fat absorption, phosphatidylcholine is involved in nerve activity. Phosphatidylinositol, on the other hand, is involved in liver metabolism, cell division, proliferation, and hormone expression. The primary building block of phosphatidylcholine, choline functions as a precursor to acetylcholine, which helps to avoid forgetfulness (Kim et al., 2021).

## 11 Conjugated linoleic acid (CLA)

CLA is a class of derivatives of unsaturated fatty acids, with isomers called 9-cis and 12-cis octadecadienoic acid, respectively, according to their geometric configurations and positions (Kim et al., 2016). Naturally occurring linoleic acid contains two double bonds, which results in the production of eight isomers, most of which are trans fatty acids. More than 98% of all CLA isomers are composed of these isomers (Hwang et al., 2021). Due to its potential as an anticancer agent, particularly in halting the development of skin cancer in mice, CLA was originally identified in fried ground beef (Kim et al., 2016). Later, studies on animals shown its efficacy against a range of cancers, including breast and colon cancers caused by different carcinogens (den Hartigh, 2019). The 9-cis, 11-trans octadecadienoic acid isomer of CLA possesses potent anticancer properties among the eight isomers (den Hartigh, 2019).

**FIGURE 9:**
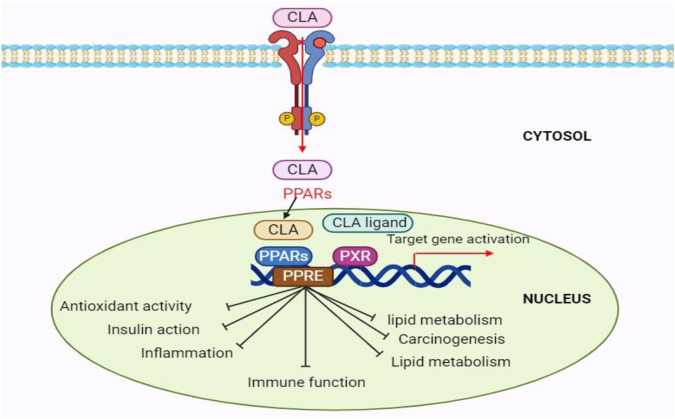
The schematic illustration of the CLA-regulated biological pathway during carcinogenic, adipose, diabatic, antioxidant, anti-inflammatory and cardiovascular diseases.

Moreover, CLA has antioxidant qualities that are comparable to butylated hydroxytoluene and better than α-tocopherol, which may protect cell membranes from damage by free radicals and have anticancer effects (Hwang et al., 2021). Furthermore, CLA inhibits atherosclerosis by causing significant decreases in triglycerides, LDL cholesterol, and total cholesterol. This effectively reduces the development of atherosclerotic plaques (Bialek et al., 2017). According to Martín-González et al. (2020), adding CLA to livestock feed promotes growth, increases lean meat content, decreases body fat, and improves feed efficiency. Commensal microorganisms, especially gut bacteria, help convert linoleic acid into CLA in animal meat or milk (Di Rienzi et al., 2018). The control of adipocyte differentiation, insulin resistance, lipid metabolism, carcinogenesis, inflammation, and immunological functions is linked to the activation of CLA-mediated CYP7A (Figure 9).

## 12 Pinitol

Pinitol, also known as D-Pinitol or 3-O-methyl-D-chiro-inositol, is a naturally occurring blood sugar regulator that may be found in pine needles and legumes. It is commonly utilized in several cultures’ traditional diabetes treatments. A methyl group is attached to the third carbon of chiro-inositol, a structural isomer of myo-inositol, via an ether bond (Li et al., 2021). By removing the methyl group from the third carbon, gastric acid causes pinitol to change into chiro-inositol (Dumschott et al., 2019). After entering the bloodstream, chiro-inositol participates in insulin and galactosamine signaling, which supports healthy energy metabolism (Bevilacqua and Bizzarri, 2018). Low chiro-inositol concentrations are seen in diabetics who have poor glucose tolerance or insulin resistance; conversely, artificial chiro-inositol treatment lowers insulin resistance, indicating improved sugar metabolism and blood sugar management (Morley et al., 2017). As a result, swallowed pinitol might function similarly to restore normalcy in the metabolism of sugar (Sánchez-Hidalgo et al., 2021). Notably, patients with type 2 diabetes have used pinitol as an oral hypoglycemic medication to control their blood sugar levels.

**FIGURE 10:**
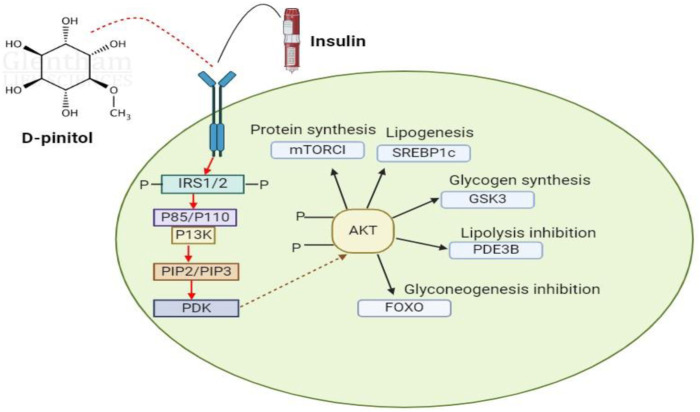
This schematic diagram show that D-pinitol have some pharmaceutical and therapeutic potential against cancer, diabetes, anti-cholesteric effects and anti-inflammatory properties.

Because of its antioxidative action on nitric oxide-mediated signaling, D-pinitol (DP) has been shown to be able to prevent diabetes-induced endothelial rupture in cardiovascular artery arteries. Uncertainty surrounds the mechanism underlying DP’s antihyperglycemic effects. The estimated antihyperglycemic action of DP is depicted in Figure 10. Plant treatments are becoming more and more popular in the field of DP-associated pharmacology due to their pharmacobiological properties, especially their anti-inflammatory and antioxidative qualities. The various *in vivo* and *in vitro* actions of pinitol help to avoid and lessen oxidative and inflammatory diseases (Antonowski et al., 2019).

Translocation of GLUT4 from endoplasmic reticulum to plasma membrane of mainly skeletol muscle is considerd the major target for insulin to maintain glucose levels. Both *in vitro* and *in vivo* experiments proved that pinitol increases the glucose uptake along wth GLUT4 translocation to the membrane. GLUT 4 due to its sensitivity to insulin plays important role in the glucose homeostasis by transporting to skeletal muscles (Ishiki and Klip, 2005). It is reported that p13K/Akt signaling pathway is mainly involved in this transportation and diabetes management by phosphatase signaling cascade (Dang et al., 2010). Therefore, D-pinitol is proved to have reduced the plasma glucose levels. It is also imperative to note that this P13K/K signaling pathway is also implicated in variety of other disease pathways like cancer, cardiovascular disorders and other neurological diseases, which are also regulated by D-pinitol by effectively reducing the concentration of blood glucose through synthesis of glycogen (Dang et al., 2010).

## 13 Therapeutic potential of soybean

This diverse array of bioactive compounds in soybean make it a good candidate to be used for various diseases. Following are some major diseases for which soybean bioactive have been used major diseases.

### 13.1 Cancer

Cancer is an abnormal cell growth that can either stay in one place inside the body or spread to other parts of it (Sanjukta and Rai, 2016). According to a study by Garg et al. (2016). From the functional viewpoint and bioactive components of soybean, dietary factors—such as food types, diversity, portion sizes, preparation techniques, and overall caloric balance—are responsible for one-third of all cancer cases. It is therefore still essential to find the bioactive substances that can stop or slow the growth of cancer cells to promote a healthy and high-quality life (Bhandari, 2014). The idea that high soy intake is linked to significantly lower incidence of cardiovascular disease and cancer is supported by a substantial body of research (Nagata et al., 2017). Still, because of their purported anticancer properties, the majority of the data points to soy isoflavones, with genistein being the key chemical of interest (Kim et al., 2014). As a well-known tyrosine kinase inhibitor (Singh et al., 2019), genistein has been shown to activate transcription factors, such as estrogen receptors, and to help induce the expression of genes in breast cancer cells (Banerjee et al., 2008). One of the suggested mechanisms involves the inhibition of topoisomerase and angiogenesis through the suppression of cell division and the disruption of the extracellular matrix degradation that envelops the formation of arteries and tumors (Singh et al., 2019; Varinska et al., 2015). Isoflavones will therefore scavenge against oxidants implicated in carcinogenesis in their role as an anticancer agent. Because genistein binds and adheres to estrogen receptor (ER) isoforms, activates peroxisome proliferator-activated receptors (PPARs), has epigenetic and genome-wide effects, and induces apoptosis, it is known to exhibit mild estrogenic action, which may inhibit the growth of cancer cells (Chen and Chien, 2014; Fürst et al., 2012). A case-control research has shown that a high soy intake during youth is associated with a lower risk of breast cancer in adulthood (Shu et al., 2001). Miso (fermented soybean paste) was fed to F344/DuCrj rats, and despite miso having 2.2% sodium chloride (NaCl) by weight, there were less precancerous colonic crypts (Masaoka et al., 1998). Rats were given a diet containing 2.2% or 4.4% wt/wt of NaCl but no miso showed no difference in the number of aberrant crypt foci as compared to the untreated controls, who were fed neither miso nor NaCl (Masaoka et al., 2000). However, a later study found no evidence that eating soy foods such as boiled beans, miso soup, and tofu protected against cancer (specifically, breast cancer) (Nishio et al., 2007). A study on soy consumption and Chinese women stands in stark contrast to this. Consuming soy has been shown in the study to have anti-inflammatory properties (Wu et al., 2012; Yan et al., 2010) a further meta-analysis also revealed a lower incidence of colon cancer (Ryan-Borchers et al., 2006). Study examined how soy isoflavones affected postmenopausal women’s immune systems. According to the authors, the administration of isoflavone resulted in a decrease in the plasma concentration (*P* < 0.5) and an increase in the B cell population (*P* < 0.05) of 8-hydroxy-2-deoxy-guanosine, an oxidative marker of DNA damage. The scientists came to the conclusion that giving postmenopausal women two soy capsules, each containing 235 mg of soy extract and 17.5 mg of isoflavones, over a period of 12 weeks (total dose of isoflavones: 70 mg/day) increased the number of B cells and showed promise in preventing DNA damage. However, after using soy supplements to alleviate menopausal symptoms, patients with early-stage breast cancer showed no discernible improvement in menopausal symptom scores (MacGregor et al., 2005). But soy peptides have also been supported in a variety of experimental models for their potential to aid in the treatment of a wide range of cancer types, including breast and prostate cancers, which are particularly interesting due to their sensitivity to sex steroid hormones (Lule et al., 2015; Pabona et al., 2013; Singh et al., 2014). Subsequent research has shown that lunasin and Bowman-Birk inhibitor (BBI), two minor 2S fraction of soy proteins, are linked to the majority of anticancer soy peptides (Lule et al., 2015; Pabona et al., 2013; Singh et al., 2014).

One well-known type of serine protease inhibitor found in large quantities in legumes, particularly soybeans, is called Bowman-Birk inhibitor. They have long been thought of as antinutrients that interfere with the process of normally breaking down protein into amino acids because they can block the activities of chymotrypsin and trypsin. As a result, they are invariably rendered inactive during the manufacturing of soy products, including soy milk and animal feed. Numerous species, including humans, have demonstrated its anticarcinogenic properties, as have tissue types like the liver, colon, breast, prostate, oesophagus, and so on (Chen et al., 2014). On HT-29 colon cancer lines, Bowman-Birk inhibitors had antiproliferative properties (Clemente et al., 2012; Fereidunian et al., 2014) as well as in animal models of carcinogenesis, by means of their effects on endogenous proteases and by the development of endoplasmic reticulum stress-mediated apoptosis, or cell death, in response to endoplasmic reticulum stress (Clemente and del Carmen Arques, 2014). Therefore, following both proteasomal inhibition and antiangiogenesis, ROS-induced mitochondrial damage triggers apoptosis, which is the mechanism by which Bowman-Birk inhibitors achieve their anticancer action (Cruz-Huerta et al., 2015; Fereidunian et al., 2014; Souza et al., 2014). The Tagalog word “Lunas,” which means “for a cure,” is where the term “lunasin” originated. The Gm2S-1 gene encodes a distinct 43-amino acid peptide sequence that is generated from soy and is bioactive.

It has been demonstrated that (SKWQHQQDSCRKQKQGVNLTPCEKHIMEKIQGRGDDDDDDDDD) functions as a chemopreventive peptide in both *in vitro* and *in vivo* settings (Chatterjee et al., 2018; Lule et al., 2015) by interacting with non-acetylated H3 and H4 histones, which in turn prevents acetylation and so expresses its anticarcinogenic action (Singh et al., 2014; Wang and De Mejia, 2005). According to reports, lausenin can be found in soy products such as tempeh, su-jae, tofu, soymilk, and soy newborn formula. It has also been proposed that lausenin can prevent skin, colon, breast, prostate, and liver cancers in animal models and cell cultures (Sanjukta and Rai, 2016). According to a study using a cell culture model, varying gene expression in response to lunasin administration with and without lipopolysaccharide stimulation influences diverse biological activities and its signaling pathways, which are not dependent on histone acetylation (Dia and De Mejia, 2011). Moreover, lunasin enhanced the tumoricidal activity of natural killer cells in both *in vitro* and *in vivo* cancer models by collaborating with immunostimulatory cytokines in immunotherapy for lymphoma (Chang et al., 2014). A review was conducted on lunasin’s ability to protect human HepG2 cells against oxidative damage. The author noted that when carcinogens are not present, the cellular morphology does not change. Therefore, adding lunasin did not directly affect the cellular morphology, but it did hinder the transformation of the cells when carcinogens are present. Nevertheless, in human HepG2 cells exposed to oxidative stress generated by tert-butyl hydroperoxide, Lunasin showed chemoprotective activity once more (Fernández-Tomé et al., 2014).

### 13.2 Antidiabetic and antiobesity effects

Type I diabetes, or insulin-dependent diabetes, occurs when the pancreas is unable to secrete insulin, and type II diabetes, or non-insulin-dependent diabetes, occurs when there is an imbalance between the absorption of blood sugar and insulin secretion. Diabetes is a well-known metabolic disease that is characterised by an increased level of blood sugar (Hamid et al., 2015). Type II diabetes in particular is a global epidemic that is affecting nearly every aspect of our society. By lowering blood glucose, soy protein has demonstrated a crucial role in diabetes. Bioactive chemicals derived from soy are thought to regulate the metabolism of fat and glucose by interacting with oestrogen receptors and appearing to have an antidiabetic impact (Garg et al., 2016; Mohamed, 2014). Obesity is the medical term for the state in which extra body fat has accumulated to the point where it could be harmful to health. Insulin resistance and type II diabetes are frequently associated with obesity and hyperlipidemia. Therefore, all soy bioactive components with hypolipidemic properties can also exhibit antidiabetic and antiobesity properties, and their efficacy has been shown in many animal models (Chatterjee et al., 2018). Triglycerides may be less likely to accumulate in the liver as a result of soy protein (Hunter and Hegele, 2017). Leu-Pro-Gly-Pro and Pro-Tyr-Pro-Arg, two peptides found in soybean glycinin protein, have shown a significant anti-obesity potential (Singh et al., 2014). By activating PPAR, a crucial transcription factor in controlling the expression of genes during glucose homeostasis, fatty acid oxidation, and lipid metabolism, soy protein can promote insulin resistance and lipid levels. Using an *in vitro* model, (Yang et al., 2011) investigated the antiobesity impact of black soy peptides. In comparison to mice fed a high-fat diet without black soybean (22.6 g), the authors observed that rats fed with both a high-fat diet and black soybean peptides ((2, 5, or 10%) for 13 weeks) gained less body weight. Although their exact mode of action is unknown, soy-derived isoflavones have been demonstrated to be useful in treating type II diabetes mellitus by reducing blood glucose levels (hyperglycemia) (Niamnuy et al., 2011). They also have antioxidant and α-glucosidase inhibitory properties. It has been demonstrated that the hypocholesterolaemic soy-derived peptides (IAVPGEVA, LPYP, and IAVPTGVA) enhance glucose metabolism by promoting glucose uptake via glucose transporters type 1 and type 4, or GLUT-1 and GLUT-4, in a hepatic cell (Lammi et al., 2015; Tsou et al., 2012). Furthermore, studies conducted *in vitro* and *in silico* have demonstrated the effectiveness of soy-derived peptides (IAVPTGVA) as a serine exopeptidase inhibitor, dipeptidyl peptidase-4 (DPP-4). In order to maintain glucose homeostasis, DPP-4 stimulates the hydrolysis of two essential polypeptides: an insulinotropic polypeptide that is glucose-dependent and glucagon-like (Lammi et al., 2016). A meta-analysis investigation on the impact of soy isoflavone supplementation on non-Asian postmenopausal women was carried out by Yang et al. (2011). According to the studies, supplementing with soy isoflavones may help lower body weight, moderate blood sugar levels, and regulate insulin levels in plasma. Similar to this, a study on type II diabetic women that fed bread (120 g) fortified with soybean flour—that is, replacing 30% of the wheat flour with soybean flour—for 6 weeks found no discernible impact on the profile (Moghaddam et al., 2014).

### 13.3 Hypocholesterolaemic and antiatherosclerosis effects

The condition known as hypercholesterolaemia, or high cholesterol, refers to elevated blood cholesterol levels. Consequently, there are higher than normal blood lipid and bad cholesterol (low-density lipoproteins, or LDL) levels. This is a well-known risk factor for coronary heart disease (CHD), which is one of the main causes of death in the west and globally (Messina, 2016; Singh et al., 2014). It has been proposed that cholesterol functions as a molecular pattern linked to harm when it activates the NLRP3 inflammasome, (Duewell et al., 2010; Progatzky et al., 2014), which promotes colitis and colorectal cancer (Du et al., 2016). Even while dietary proteins, such as those found in soy foods, are thought to help stimulate the effect of blood cholesterol concentrations, there is still debate regarding the cholesterol-lowering (or hypocholesterolemic) effects of soy protein (Messina, 2016; Singh et al., 2014). Nonetheless, a multitude of studies support soy protein as the primary nutrient accountable and refute soy isoflavones (Messina, 2016). Studies on animals and cell cultures revealed that isoflavones had a beneficial effect on reducing atherosclerosis and lowering cholesterol levels in (LDL) low-density lipoprotein by preventing oxidation and increasing (HDL) high-density lipoprotein in the body. In (Millar et al., 2017) Numerous findings have confirmed the hypocholesterolaemic activity of dietary proteins and their bioactive peptides, including modifications to bile acid secretion, alterations in liver cholesterol metabolism, hormonal effects, and control of cholesterol receptors (Singh et al., 2014). It has been demonstrated that cholesterol metabolism is essential for increasing T-cells’ adaptive immunological response (Tall and Yvan-Charvet, 2015). Particularly, it has been demonstrated that oxysterols, the byproduct of cholesterol metabolism, block the synthesis of cholesterol by binding to the liver’s X receptor β (LXRβ). Oxysterols become sulphated upon T-cell activation, which causes them to no longer cling to LXRβ. Therefore, to boost proliferation in the acquired immune response, T-cell cholesterol metabolism promotes cholesterol production (Bensinger et al., 2008; Kloudova et al., 2017). Due to their potential as a substitute for traditional hormone replacement treatment, their capacity to lower cholesterol, and their antiatherogenic health effects, soy protein, and isoflavones garnered international attention and were extensively discussed in 1995 (Messina, 2010). Subsequent research revealed that consuming 25% soy protein on a daily basis could help lower blood levels of triglycerides, low-density lipoprotein (LDL), and serum cholesterol by 12.9%, 10.5%, and 9.2%, respectively (Bhoite et al., 2021). Another meta-analysis showed that while isoflavone phytoestrogens had little effect on patients with normal serum cholesterol concentrations, they did lower the plasma cholesterol concentrations of those with increased levels (Wang et al., 2013). The effects of soy meals on blood cholesterol levels after oral administration to both people and animals were taken into consideration while evaluating their benefits on cardiovascular illnesses (Bhoite et al., 2021). As a result, these dietary proteins undergo protease breakdown in the stomach, releasing bioactive peptides that may lower cholesterol levels. Similarly, the American Diabetes Association (ADA) suggests that consuming 26–50 g of soy protein daily (instead of animal protein) for 5 weeks would be sufficient to prevent and treat cardiovascular disease (CVD) (Bhoite et al., 2021; Koutelidakis and Dimou, 2016). According to a study by Torre-Villalvazo et al. (2009), dietary soy protein (i.e., with an adjusted protein concentration purity of 90.6% casein, 86% soy protein) can reduce cardiac triglyceride and cholesterol concentrations as well as suppress cardiac ceramide concentrations by lowering lipid accumulation and inhibiting the expression of serine palmitoyltransferase, the key enzyme in sphingolipid biosynthesis, in the hearts of obese mice. Therefore, consuming soy protein could be a helpful dietary treatment approach to stop lipotoxic cardiomyopathy. A major storage protein found in soybeans, 7S globulin, was shown in an *in vivo* investigation involving rats to drastically reduce plasma cholesterol content by 35% (Lule et al., 2015). Contradictory findings revealed that nonprotein fraction, isoflavones, and saponins were unable to demonstrate any beneficial effects in lowering cholesterol (Adams et al., 2004). This was clarified when rats were given LPYPR derived from soybean glycinin subunit at a dose of 50 mg/kg without isoflavones for 2 days, and the rats’ serum total and LDL cholesterol levels decreased by 25% (Chatterjee et al., 2018). A tetrapeptide (LPYP) from the soy protein glycinin was used in a later investigation to demonstrate a hypercholesterolaemic effect (Kwon et al., 2002). Additionally, the Mangano et al. (2013) study clarified that while the administration of soy protein (18 g/d) and isoflavone tablets (105 mg/d isoflavone aglycone equivalents) to 131 healthy, ambulatory older women was effective in reducing inflammatory markers (IL-6), it did not improve serum lipid levels, which are thought to be a critical risk factor in coronary heart disease (CHD). In a similar vein, (Wang et al., 2013) proposed a theory that soy isoflavone, specifically genistein, is implicated in the pathophysiology of Kawasaki disease, which is associated with children aged 5 and under and causes inflammation of the blood arteries throughout the body, especially in the heart. This peptide functioned as a competitive indicator of 3-hydroxy-3-methylglutaryl CoA reductase, or HMG-CoA reductase, which is the primary enzyme that limits the rate of cholesterol biosynthesis (Chatterjee et al., 2018; Pak, Koo et al., 2012). A further animal model study found that feeding pigs a lunasin-enriched soy extract in addition to casein reduced their levels of low-density lipoprotein (LDL) cholesterol when compared to the group that only received a casein diet, which indicated a faulty LDL receptor gene (Galvez, 2012). In the last few years, peptide extract, protein complexes, and dietary supplements have been added to lunasin-enriched soy extract to create a variety of functional foods. Dietary supplements made from soybeans, such as Lunasin XPR^®^ (peptide extract), LunaSoyTM (protein complex), and LunaRich^®^, are utilized commercially as potential ingredients in functional foods that lower cholesterol and as dietary supplements for heart disease and general cellular health (Lule et al., 2015; Udenigwe and Aluko, 2011). It is important to remember, nevertheless, that studies on soy protein’s ability to lower cholesterol have been conducted using *in vivo* models and cell lines. Additionally, clinical trials must be conducted in order to determine the protein’s safety and effectiveness in therapeutic applications (Lule et al., 2015).

### 13.4 Antihypertension effects

The third most significant risk factor for serious health issues, including early mortality, globally is hypertension, or high blood pressure, which is a result of the angiotensin-converting enzyme (ACE), a crucial component in blood pressure management (Zhong and Schleifenbaum, 2019). Angiotensin-I converting enzyme (ACE), the first step in the renin-angiotensin system, is inhibited by antihypertensive or ACE inhibitory peptides. This affects the negative feedback effects of angiotensin II and controls blood pressure and fluid balance in the body. By cleaving the dipeptide at the C-terminus, the dipeptidyl carboxypeptidase activities of ACE transform the dormant decapeptide angiotensin I into the active vasoconstricting octapeptide angiotensin II, which raises blood pressure (De Mejia and Ben, 2006; Natesh et al., 2003). It is proposed that one of the most important treatment strategies for managing hypertension is ACE inhibition. In addition, a number of risk factors, including heart failure, myocardial infarction, coronary heart disease, and stroke, are thought to be the fundamental cause of hypertension (Singh et al., 2019). Notably, a study on the ACE-Inhibitory activity of soy protein isolates in the prevention of hypertension in an *in vivo* model showed that, when 25 g of non-soy protein were substituted with 25 g of soy nuts (i.e., soy nuts containing 25 g of soy protein and 101 mg of aglycone isoflavones), hypertensive women and normotensive postmenopausal women’s blood pressure increased, all of which were attributed to the cardioprotective effect (Margatan et al., 2013; Welty et al., 2007). Using *in vitro* and *in silico* models, (Zhao et al., 2019) investigated new ACE inhibitors generated from soybean proteins. Despite being a strong ACE inhibitory peptide, dimethylglycine (DMG) had no deleterious effect on HEK-293 cells. The outcomes of the molecular docking analysis showed that DMG and ACE’s activities (Ala354, Glu384, Gln281, His353, His513, Lys511, and Tyr520) interacted well. According to the authors, DMG exhibited strong action compared to ACE, with an IC50 value of 3.95 ± 0.11 mM. Using human platelets that had been separated, black soybean extract also demonstrated a potent inhibitory effect on platelet aggregation brought on by collagen in an *in vitro* model (Kim et al., 2011). The review also covered the investigation on the functional characteristics and antihypertensive-inhibitory activity of produced soy whey protein and fractions. Using ultrafiltration, the whey proteins were divided into several fractions; the unfractionated whey protein had the strongest inhibitory activity of the angiotensin-I converting enzyme. Once more, the whey protein fraction (>50 kDa) showed superior solubility, stability, and emulsion activity; in contrast, the unfractionated sample showed the highest percentage of nineteen (19%) of angiotensin-I converting enzyme inhibition (Lassissi et al., 2014). About 33 peptides with angiotensin-I converting enzyme (ACE) inhibitory action were found in *Lactobacillus* plantarum strain C2 fermented soymilk, according to a study by Singh and Vij (2017); Acharjee et al. (2015) studied confirmed that postmenopausal women who were categorized by metabolic syndrome status experienced a greater decrease in blood pressure, inflammatory markers, and molecule adhesion after receiving soy protein (25 g) and isoflavones (101 mg) supplements. Additionally, a study found that consuming foods high in soy fibre, such as biscuits supplemented with soy fibre (about 100 g/day for 12 weeks), resulted in a significant reduction in body weight and lipid content in an adult who was overweight or obese. These conditions are known to be symptoms of hypertension, obesity, and hyperlipidemia, among other conditions (Hu et al., 2013). Additional research on animals revealed that soybeans may have a preventive role against cardiovascular disease when the foetus is exposed to isoflavones through the mother’s diet (Bonacasa et al., 2011). In contrast to chemosynthetic pharmaceuticals, ACE-inhibitory peptides derived from plants and animals may prove to be a viable substitute due to their safety, growing demand, and cost-effectiveness (Singh et al., 2014).

## 14 Conclusion

Soybeans have considerable health and therapeutic potential due to their varied range of bioactive chemicals. A wide variety of bioactive substances, including as proteins, saponins, carotenoids, phytosterols, isoflavones, and tocopherols, are found in soybeans and have been shown to have major health advantages. As phytoestrogens, isoflavones have antioxidant properties and lower the risk of cancer. Phenolic substances are involved in the regulation of estrogen and antioxidant activity. Saponins have anticancer properties and help lower cholesterol. Once thought to be antinutritional, protease inhibitors now exhibit anticancer and antioxidant qualities. Phytic acid lowers cholesterol and possesses antioxidant properties, albeit impeding the absorption of minerals. Fiber and soy oligosaccharides support intestinal health. Lecithin promotes brain health, lowers cholesterol, and facilitates the absorption of nutrients. CLA lowers cholesterol and has anticancer properties; pinitol controls blood sugar. The novel medical and pharmaceutical uses of soy bioactive components have great promise for improving human health and well-being as long as research is conducted. Including soy in diet may be a natural, all-encompassing way to improve general health and ward against a number of chronic illnesses.

## 15 Future perspective

The use of soy in therapeutic applications and health enhancement shows significant promise for the future. Researchers are discovering special ingredients in soy called bioactive compounds that have amazing health benefits. These substances may be used to treat and prevent a number of illnesses, including diabetes, heart problems, and some forms of cancer. Researchers anticipate using these bioactive molecules for other purposes in the future, recommending different courses of action for people depending on their particular needs. Furthermore, it's possible that these soy chemicals will be included into better and new medications, administered using cutting-edge technology to provide better outcomes with fewer adverse effects. As we move forward, there are a lot of opportunities for developing better and more effective solutions for general well-being thanks to the potential of soy bioactive components.

Several studies have shown how technological developments have led to the global spread of genetically modified (GM) crops. Genetically engineered soybeans that are widely planted either appear on our plates in a variety of culinary forms or are blended with other components to make processed foods. Regarding the impact of these genetically engineered crops and commodities on health, opinions vary. Concerns about the environment and public health have led to calls for stringent laws that are meant to lessen risks. Notably, cutting edge genetic engineering methods like genome editing have lately been used to develop novel ways to gene correction that do not rely on conventional genetic modification techniques. This new generation of genetically modified crops is being brought about by these technologies in conjunction with sophisticated biotechnological instruments. It is expected that traditional crop breeding will eventually converge with biotechnology, genetic engineering, and molecular breeding technologies due to the world’s growing population and issues like food scarcity, climate change, and pollution. With the use of soy bioactive components in cutting-edge drug delivery systems, nanotechnology is set to play a critical role in maximizing pharmaceutical efficacy and reducing side effects. Furthermore, nanotechnology can be used to solve issues with the development of highly functional health foods.
